# Rare diseases and orphan drugs: 500 years ago

**DOI:** 10.1186/s13023-015-0353-3

**Published:** 2015-12-21

**Authors:** Marc M. Dooms

**Affiliations:** Universitaire Ziekenhuizen Leuven, Leuven, Belgium

## Abstract

In 1581 Rembert Dodoens wrote “Medicinalium observationum exempla rara, recognita et aucta” a Latin book about the diagnosis and treatment of disorders with a low prevalence.

The “orphan drug-movement” is believed to have been initiated only recently with the Orphan Drug Act of 1983 in the United States of America, which was intended to stimulate research and commercialization of medicinal products (“orphan drugs”) intended for the in-vivo diagnosis, prevention and treatment of diseases with a low prevalence (“rare diseases”). But was there any interest in disorders with a low prevalence before last century?

Rembert Dodoens was a Flemish physician also known as Rembertus Dodonaeus, who was born in Mechelen (now Belgium) on the 29th of June 1517. He did his studies at the Collegium Trilingue (founded by Desiderius Erasmus in 1518) in Leuven as did Andreas Vesalius and at the University of Leuven where he graduated as a physician in 1535. After his studies he established himself as a physician in his hometown Mechelen and turned down positions as professor in medicine at the University of Leuven and as court physician of King Philip II of Spain. After extensive travelling, he joined the Faculty of Medicine at Leiden University as a professor in 1582 where he died on the 10th of March 1585. His most famous book is his *Cruydenboeck* (“herb book”), which is the the first pharmacopoeia of medicinal herbs. In 1581, he wrote *Medicinalium observationum exempla rara*. Two copies of this book written in Latin are in the Tabularium of the central library of the University in Leuven: the first edition (1581, 397 pages) is catalogued as DPA510 and the second (1585, printed by the famous Christopher Plantijn in Antwerp) is catalogued as 7A875. In 1543, Andreas Vesalius, his schoolmate in Leuven, published the revolutionary seven-volume work on human anatomy, *De Humani Corporis Fabrica*, based on dissections of human bodies. In 1546, Hieronymus Fracastorius, a colleague of Vesalius in Padua, suggested in his *De Contagione et contagiosis Morbis* that epidemic diseases could be caused by the transfer of tiny particles.

*Medicinalium observationum exempla rara, recognita et aucta. Accessere et alia quaedam, quorum elenchum pagina post praefationem exhibit* by Rembert Dodoens medici Caesarei along with Valesco de Tarenta, Alexander Benedictus, Antonio Benivieni, Maternus Cholinus, Mathias Cornax, Achilles Pirmin Gasser and Gilles de Hertoghe. After a general introduction and a list of cited authors (including Vesalius and Fracastorius), Rembert Dodoens gives extensive list of some 200 rare diseases in the 16th century such as *Aneurisma*, *Calculus in vesica* (stone in the bladder), *Catalepsis* (seizure), Diabetes, *Dysenteria*, *Gemini pueri* (twins), *Lapides in vessica fellis* (gall stones), *Mania cum Melancholia affinitatem habet* (mania with melancholia) *Scorbutus*, *Tetanos*, *Vermis in vesica* (worms in the bladder) and *Vomitus sanguinis* (vomiting blood). These are all macroscopic (“*de visu et de manu*”) or organoleptic observations as Antoni Van Leeuwenhoek (1632–1723) started to use a microscope in cell- and microbiology only in 1674. Also Syndromes Without a Name (SWAN) are mentioned such as *Memoria amisa ac recuperate* (“he lost his memory and recovered it”), *Respirandi difficultas* (difficulty breathing) and *De pedis tumore quem Arabes Elephantiam appellant*” (swelling of the feet called elephantiasis by the Arabs).

For surgical procedures Dodoens called upon his local barber-surgeons (*amputatio*) or stone-cutters (*lithotomia*). For pharmacological treatment Dodoens referred to his own *Cruydenboeck* (Book of Herbs, Mechelen, 1554) with “*Plaetse, Tijt, Naem, Natuere, Kracht ende Werckinghe*” (growing place and time, name, identification, pharmacological activity) of 942 plants. Operating procedures for the production of his “orphan drugs” from plant material (*bolus, confectio, decoctum, electuarium, hostia, pilula, potio, pulvis, syrupus, trochiscus* for internal use and *balneum, cauterium, clysterium, collyrium, emplastrum, gargarisma, lotio, unguentum* for external use) are given at the end of some monographs.

The standardization of these compounding procedures by local pharmacists continued further with the publication of several city and (supra)national pharmacopoeias. Only at the end of the 19th century the pharmaceutical industry will take over the manufacturing of medications. In 2012, the European Medical Agency accorded a Market Authorization to the orphan drug *Nexobrid*, a purified extract from pineapple (*Ananas comosus*). Colchicine, the active ingredient of *Colchicum autumnale* (Cruydenboeck Part 3, Plant 40), is still used today off-label for the treatment of ultra-rare auto-inflammatory diseases. Off-label use was not mentioned as the FDA required the first package insert only in 1968. Home therapy was done by pharmacists administering clysters at home.

Throughout the Middle Ages, to be diagnosed with a (rare) disease had major social (“stigmatization”) and medicinal implications for the individual. Some communities, knowing the importance of an accurate diagnosis, established multidisciplinary groups (“expert centers”) to review suspected cases. Representatives of the church, physicians and people with the disease (“patients’ representatives”) were typically members of these groups. Cousin marriage was common at that time in Europe, which most probably resulted in multiple genetic diseases. These disorders could not yet be diagnosed on DNA as James Watson and Francis Crick discovered the double helix only in 1953. The first description of a rare disease attributed to inheritance (alkaptonuria) was by Archibald Garrod in 1902 on the basis of proteins not DNA. Children were born at home from teenage house-wives sometimes with the help of a midwife following the guidelines of Eucharius Rhodion’s *Der Rosengarten* (1513). Long-time breastfeeding by the mother or by a wet nurse was general practice. Infanticide became exceptional in the 16th century, and unwanted children were left at the door of church or abbey, and the clergy was assumed to take care of their upbringing.

William Harvey (1578–1657) was the first to describe correctly the systemic circulation of blood pumped to the brain and body by the heart. He is also famous for his quote on rare diseases: *Nature is nowhere accustomed more openly to display her secret mysteries than in cases where she shows traces of her workings apart from the beaten path; nor is there any better way to advance the proper practice of medicine than to give our minds to the discovery of the usual law of nature by the careful investigation of cases of rarer forms of disease. For it has been found in almost all things, that what they contain of useful or of applicable nature, is hardly perceived unless we are deprived of them, or they become deranged in some way.*

Over the last 500 years at least, there has always been interest on the part of the medical and pharmaceutical profession in the diagnosis and treatment of disorders with a low prevalence. But of course, diagnostic (uroscopy as the lab test) and surgical procedures have changed tremendously over time. Life-style changes (scorbutus/vitamin C; dysenteria/better quality of drinking water) and vaccinations (tetanos/Clostridium) have almost eliminated some and a better understanding of the diseases has led to prevention and better (intravenous) therapy. Compounding medications following Standard Operating Procedures and Good Manufacturing Practice is still practiced today but most of the medicinal products for the prevention, diagnosis and treatment of a limited number of rare diseases are now commercially available. More basic research and randomized clinical trials are needed for the cure of patients with other rare –- and ultra-rare—diseases.
